# Eugenol-Functionalized Magnetite Nanoparticles Modulate Virulence and Persistence in *Pseudomonas aeruginosa* Clinical Strains

**DOI:** 10.3390/molecules26082189

**Published:** 2021-04-10

**Authors:** Hamzah Basil Mohammed, Sajjad Mohsin I. Rayyif, Carmen Curutiu, Alexandra Catalina Birca, Ovidiu-Cristian Oprea, Alexandru Mihai Grumezescu, Lia-Mara Ditu, Irina Gheorghe, Mariana Carmen Chifiriuc, Grigore Mihaescu, Alina-Maria Holban

**Affiliations:** 1 Microbiology & Immunology Department, Faculty of Biology, University of Bucharest, 77206 Bucharest, Romania; hamza_basil@yahoo.com (H.B.M.); sajjadmohsin13@gmail.com (S.M.I.R.); carmen.curutiu@bio.unibuc.ro (C.C.); lia-mara.ditu@bio.unibuc.ro (L.-M.D.); irina.gheorghe@bio.unibuc.ro (I.G.); carmen.chifiriuc@bio.unibuc.ro (M.C.C.); grigore.mihaescu@bio.unibuc.ro (G.M.); 2 Research Institute of the University of Bucharest—ICUB, University of Bucharest, 050657 Bucharest, Romania; grumezescu@yahoo.com; 3 Department of Science and Engineering of Oxide Materials and Nanomaterials, Faculty of Applied Chemistry and Materials Science, Politehnica University of Bucharest, 011061 Bucharest, Romania; ada_birca@yahoo.com (A.C.B.); ovidiu73@yahoo.com (O.-C.O.)

**Keywords:** virulence modulation, magnetite nanoparticles, persistent infections, biofilm

## Abstract

Efficient antibiotics to cure *Pseudomonas aeruginosa* persistent infections are currently insufficient and alternative options are needed. A promising lead is to design therapeutics able to modulate key phenotypes in microbial virulence and thus control the progression of the infectious process without selecting resistant mutants. In this study, we developed a nanostructured system based on Fe_3_O_4_ nanoparticles (NPs) and eugenol, a natural plant-compound which has been previously shown to interfere with microbial virulence when utilized in subinhibitory concentrations. The obtained functional NPs are crystalline, with a spherical shape and 10–15 nm in size. The subinhibitory concentrations (MIC 1/2) of the eugenol embedded magnetite NPs (Fe_3_O_4_@EUG) modulate key virulence phenotypes, such as attachment, biofilm formation, persister selection by ciprofloxacin, and the production of soluble enzymes. To our knowledge, this is the first report on the ability of functional magnetite NPs to modulate *P. aeruginosa* virulence and phenotypic resistance; our data highlights the potential of these bioactive nanostructures to be used as anti-pathogenic agents.

## 1. Introduction

Despite the discovery of vaccines and antibiotics, infectious diseases are still one of the main causes of death [1]. Currently, the medical field has to face a continuously growing population of multiple-drug, extended-drug, and pan-drug resistant bacteria, responsible for most difficult-to-treat infections, even more complicated by the increased tolerance of bacterial biofilms, requiring high doses of antibiotics that can be life-threatening due to their toxic effects [2]. Moreover, partially degraded or intact antibiotic molecules are released in huge amounts in waters and soil, polluting the environment while increasing the selective pressure for resistant mutants [3]. Since the continuous increase of multidrug-resistant bacteria, the focus of research has shifted towards the antimicrobial activity of other agents such as plant-derived compounds [4] or nanoparticles [5,6]. Natural products represent a great source of diverse and useful chemical compounds, which have the potential to be used in many biomedical applications, including the control of severe infections [7]. They represent a natural and ecological alternative with great potential in the design of therapeutic and prophylactic strategies [8]. Most available papers refer to the ability of these plant-derived compounds to interfere with the development and viability of bacteria [9,10]. Our previous studies have shown that certain essential oils (EOs) may also impact microbial virulence and Quorum Sensing (QS) signaling, modulating the expression of some genes involved in the main mechanisms of intercellular communication in *Staphylococcus aureus* and *Pseudomonas aeruginosa* [11]. Additionally, recent studies have reported that purified EO-derived compounds such as trans-cinnamaldehyde, eugenol, and carvacrol interfere with microbial virulence, reducing biofilm formation and modulating the expression of target virulence genes in gram-negative pathogens, such as *Campylobacter jejuni* [12].

Eugenol is the main constituent of clove (*Syzygium aromaticum*) EO. The antimicrobial activity of eugenol is mainly attributed to its phenolic structure and to the hydrophobic character of this compound, facilitating its interaction with the microbial cell envelope [13,14].

Although many natural substances have demonstrated significant antimicrobial activity, some of their properties, such as volatility, instability, and high doses required for an efficient therapy, currently limit their use in the biomedical practice [15].

A promising strategy is using nanometric vehicles to transport and release the antimicrobial agent in a controlled manner. Magnetic nanoparticles have gained a lot of attention for this kind of application, due to their advantages such as appropriate size; capacity to potentiate the antimicrobial activity of different compounds, thus decreasing the required active dose; and, most importantly, controlled release and guidance using magnetic fields [15,16]. These aspects had been exploited for applications such as medical imaging, hyperthermia, controlled drug and other bioactive molecules release, etc. [17]. Although therapeutic magnetite systems had satisfactory results from the point of view of biocompatibility and functionality, their standardization has been quite difficult to achieve; presently, only systems for medical imaging and some employed for the treatment of cancer by magnetic hyperthermia are commercially available [18].

*Pseudomonas aeruginosa* is a multidrug-resistant gram-negative opportunistic bacterium that can be found in diverse niches from plants, animals, and humans, also causing severe infections, especially in ventilated patients [19]. Due to its intrinsic resistance and the great capacity to additionally acquire resistance genes by horizontal transfer, *P. aeruginosa* infections are very difficult to treat [20,21].

This paper reports on the physico-chemical characterization and antimicrobial potential of newly developed bioactive magnetic nanoparticles functionalized with eugenol, emphasizing their effect on virulence and persistence of laboratory and clinical *P. aeruginosa* resistant strains.

## 2. Results and Discussion

### 2.1. Physico-Chemical Characterization of the NPs 

The results of X-ray diffraction studies on the obtained Fe_3_O_4_ nanoparticles demonstrated the correspondence with the characteristic diffraction interferences of magnetite. The pattern has characteristic peaks at 30.33° (220), 35.51° (311), 43.16° (440), 53.61° (422), 57.17° (511), and 62.82° (440). All interferences can be indexed using the JCPDS number 19-0629 corresponding to the magnetite [22].

**Figure 1:**
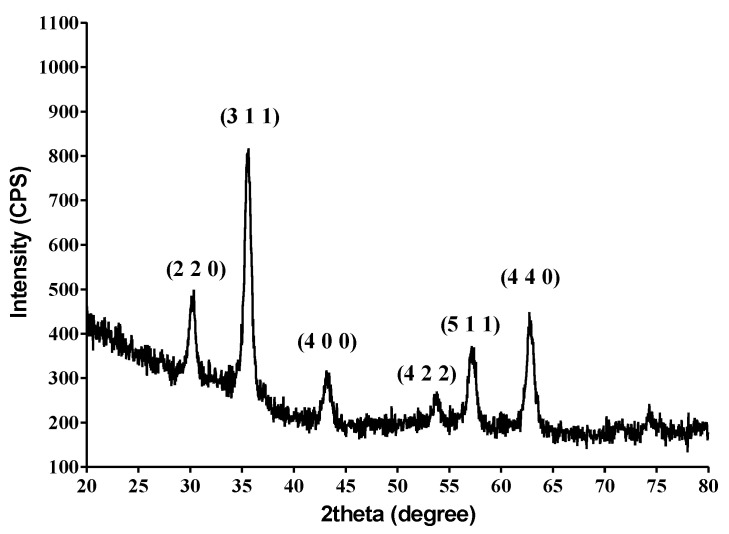
X-ray diffraction pattern of Fe_3_O_4_@EUG.

By comparing already published magnetite X-ray spectra [23,24] with the patterns resulting from the X-ray diffraction analysis of eugenol functionalized magnetite samples (presented in Figure 1), it could be observed that the presence of the antimicrobial compound eugenol did not affect the magnetite-specific crystalline structure. Due to the small amount of the therapeutic agent attached to the surface of the nanoparticles obtained, no significant decrease in the intensity of the diffraction peaks was observed.

**Figure 2:**
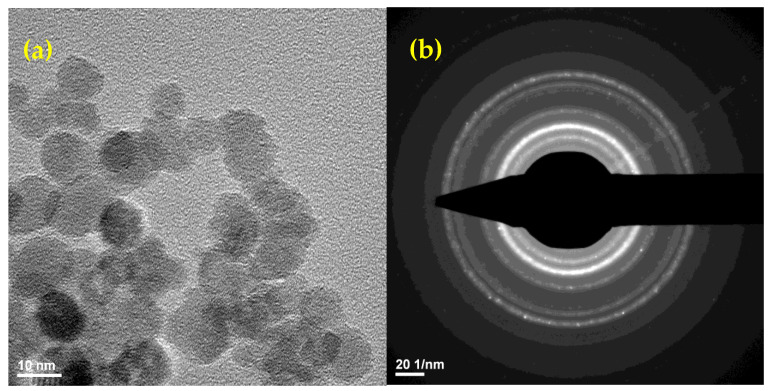

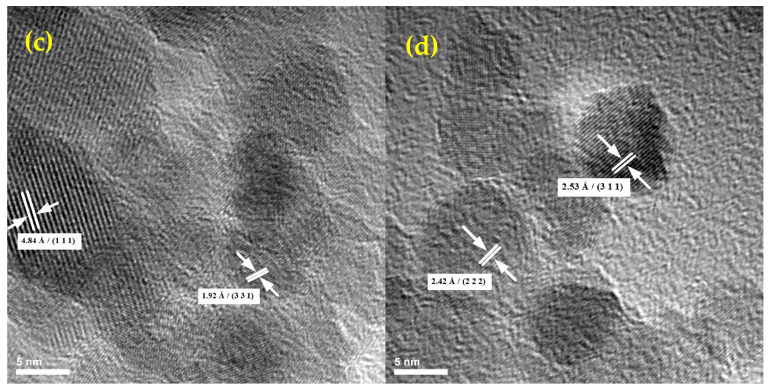
TEM (**a**), SAED (**b**) and HR-TEM (**c**,**d**) images of the obtained functionalized magnetite nanoparticles.

Regarding the structure, following the analysis of transmission electron microscopy images, a high degree of crystallinity of the nanostructures could be observed. Regarding the morphology of the obtained samples, for magnetite functionalized with the natural therapeutical agent, a small degree of agglomeration is observed, the particles of magnetite being dispersed in an organic matrix of reduced crystallinity (according to the HR-TEM images). Particle dimensions range from 10 to 15 nm on average (Figure 2).

The SAED analysis confirms the polycrystalline character of the obtained samples, with the resulting spectra allowing the identification of crystalline planes for magnetite [25]. According to the SAED measurements, the analyzed samples were polycrystalline, the only identified crystalline mineral phase being magnetite. Additionally, based on the SAED measurements, the characteristic crystallinity aspects of the studied nanoparticles were identified (Figure 2).

**Figure 3:**
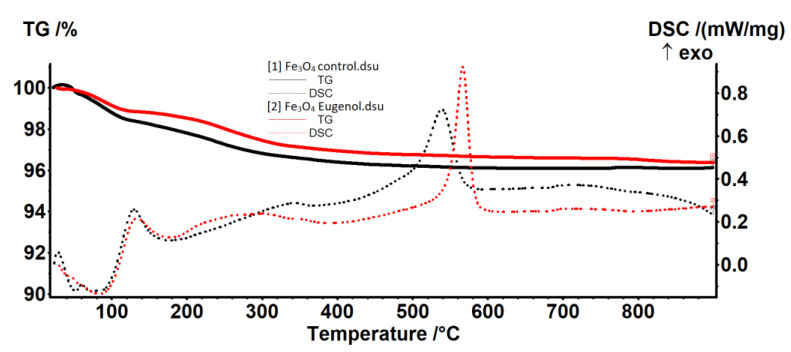
Thermogravimetry/differential scanning calorimetry (TG-DSC) results for the obtained magnetite nanoparticles.

The thermal analysis (TG-DSC) showed that the samples have a similar thermal behavior. In the temperature interval RT−120 °C, weak-bonded water molecules from the nanoparticles surface are eliminated (Figure 3).

The existence of two separate endothermic peaks at 52.3 and 73.1 °C indicate that some water molecules have a really weak interaction with Fe_3_O_4_. In the Fe_3_O_4_—eugenol sample, these weakly-bonded water molecules are replaced by some eugenol molecules, until an equilibrium is attained between interactions of Fe_3_O_4_ with water and eugenol molecules. Therefore, only one endothermic peak can be observed at 83.7 °C for the sample Fe_3_O_4_—eugenol.

In the temperature interval 120–400 °C, the oxidation of magnetite to maghemite takes place, together with the elimination of strong-bonded −OH moieties from the surface of the nanoparticles (Figure 3).

The predominant effect is exothermic (oxidation of magnetite to maghemite), but it has a weak intensity and is very broad. The peak value is at a lower temperature (299 °C) for the sample Fe_3_O_4_—eugenol, indicating quicker oxidation than in the Fe_3_O_4_ control sample (345.4 °C) (Table 1). 

After 400 °C the maghemite is transformed into hematite, a specific phase transition, without mass loss, the characteristic exothermic peak being shifted towards higher temperatures in the case of the Fe_3_O_4_-eugenol sample. Despite this temperature shift, and the change of the peak shape, the transformation of maghemite to hematite presents a similar thermal effect, the area being 123 J/g for Fe_3_O_4_-control and 122 J/g for Fe_3_O_4_-eugenol. The narrow, sharp shape of the DSC peak from 566.7 °C indicates that the Fe_3_O_4_ eugenol sample is composed of smaller, uniform-size nanoparticles (Table 1).

The residual mass for both samples is composed of hematite.

**Figure 4:**
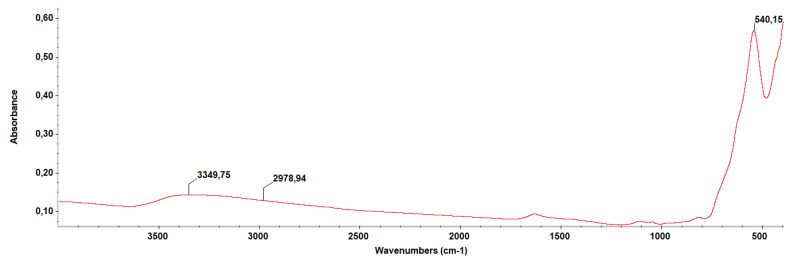
IR spectrum of Fe_3_O_4_-Eug.

The IR spectrum (Figure 4) highlights the presence of a Fe-O bond at 540 cm^−1^, and the presence of eugenol through the absorbance recorded at 2978 cm^−1^ for C-H vibration. Additionally, the OH group was observed at 3349 cm^−1^.

### 2.2. In Vitro Antimicrobial Analysis and Virulence Modulation

#### 2.2.1. Qualitative Antimicrobial Analysis—Growth Inhibition

**Figure 5:**
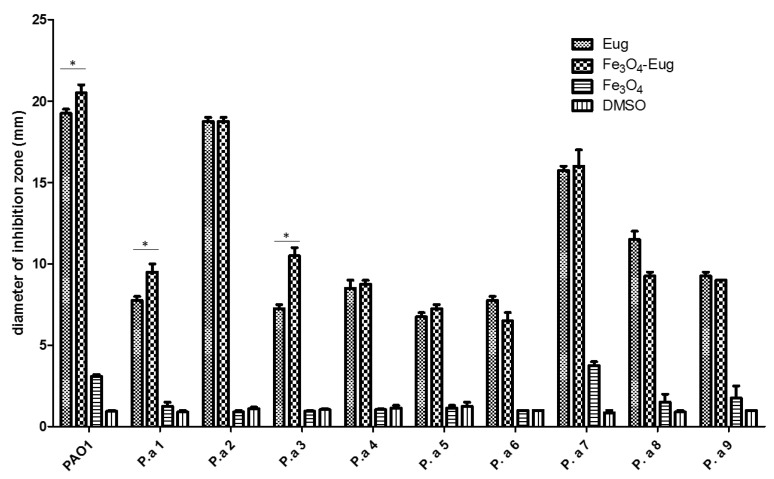
Graphic representation of growth inhibition diameters (shown in mm) obtained after the cultivation of *P. aeruginosa* strains in the presence of magnetic nanoparticles functionalized with eugenol (Fe_3_O_4_-EUG), and the controls represented by eugenol, Fe_3_O_4_ control, respectively, dimethyl sulfoxide (DMSO) solvent in which eugenol/hybrid nanosystems were diluted (one-way ANOVA, * *p* < 0.05; significant evidence of enhanced activity of the nanosystem compared to eugenol was marked only).

The evaluation of the growth inhibition results determined that the functionalized nanoparticles showed a different antimicrobial effect, depending on the tested strain. Eugenol functionalized nanoparticles exhibited a significant inhibitory effect on the growth of all tested microorganisms. As shown in Figure 5, plain eugenol and Fe_3_O_4_-eugenol inhibits the growth of laboratory PAO1 strain and of clinical strains encoded P.a 2 and 7, which are more susceptible to antibiotics compared to other clinical strains [26]. Eugenol functionalized NPs proved to be more effective in inhibiting the bacterial growth compared to the control compound (eugenol) in the case of three of the analyzed *P. aeruginosa* strains (PAO1, P.a 1, and P.a 3). In one strain, namely P.a 2, the diameter of the inhibition zone obtained for Fe_3_O_4_-eug is identical to that obtained for the eugenol control utilized at the same concentration (Figure 5). It can also be noticed that the plain magnetite NPs has a very low antimicrobial effect, observed in the case of PAO1 and P.a 7 strains, and in both cases, a synergic antimicrobial effect of magnetite nanoparticles and eugenol can be observed. The solvent (DMSO) showed no significant antimicrobial effect for the tested *P. aeruginosa* strains (Figure 5).

#### 2.2.2. Quantitative Analysis of Antimicrobial Effect—MIC Values

**Figure 6:**
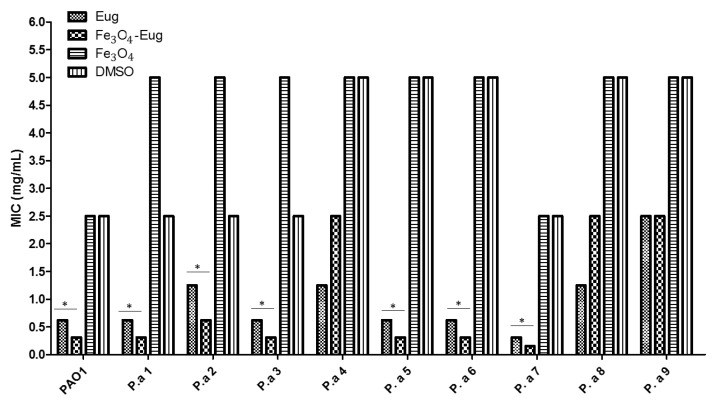
Graphic representation of MIC values represented in mg/mL (one-way ANOVA, * *p* < 0.05).

In order to quantify the antimicrobial effect of functionalized magnetic nanoparticles, a minimal inhibitory concentration (MIC) assay was performed. The results showed that the magnetite nanoparticles functionalized with eugenol exhibited reduced MIC values for most tested strains, with the MIC being under 1 mg/mL for 7 out of 10 tested *P. aeruginosa* strains. The majority of the MIC values were in the range of 0.312 to 0.625 mg/mL, about 50% lower compared to the MIC values obtained for eugenol (used as control) for the same strains (Figure 6). There were significant variations recorded between the analyzed strains, observing that for the antibiotic susceptible P.a 7 strain, the MIC values of both eugenol and functional nanosystem were reduced compared to the MIC obtained for resistant isolates. Control Fe_3_O_4_ nanoparticles (plain magnetite) as well as the DMSO control showed a weak antimicrobial effect, considering the high MIC values achieved (MIC of 2.5–5 mg/mL) (Figure 6).

#### 2.2.3. Evaluation of the Soluble Virulence Factors Production

**Figure 7:**
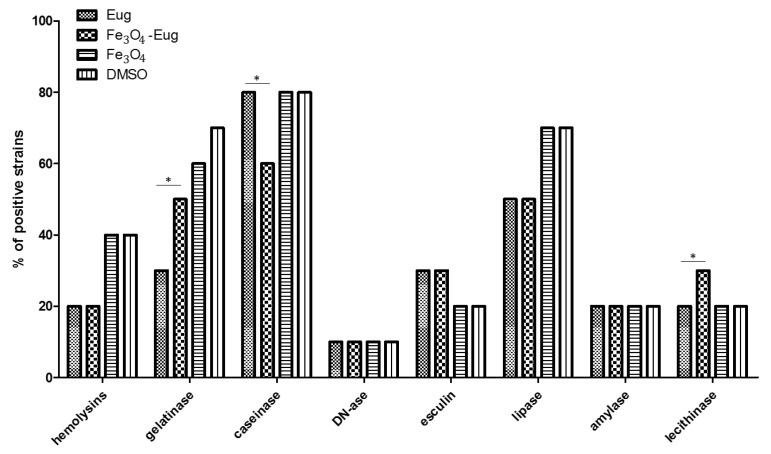
The influence of nanobiomaterials and controls on *P. aeruginosa* soluble virulence factors production. The graph represents the number of strains with the modified phenotype, expressed as a percentage (positive for the production of tested soluble virulence factors), following cultivation in the presence of the tested nanomaterials and controls (one-way ANOVA, * *p* < 0.05; significant evidence of the difference in virulence exhibited by eugenol and magnetite-eugenol nanoparticles).

In order to analyze the effect of tested nanobiomaterials on the ability of *P. aeruginosa* to produce soluble virulence factors, a method based on cultivation on special media containing different substrata was used. Evidence of soluble virulence factors release was achieved either directly by assessing changes in color or transparency or after adding some reagents to the medium in order to observe immediate biochemical reactions developed near the microbial colonies [27]. The obtained results showed that nanomaterials based on magnetite functionalized with eugenol presented different microbial virulence modulatory effects, depending on the *P*. *aeruginosa* tested strain. The modulated phenotypes were represented by hemolysins, gelatinases, and lipases, the production of these factors being diminished by the functional nanoparticles and, in few cases, by the plain eugenol. The DNA-se and amylase production was not affected by the tested variants used in the study (Figure 7).

#### 2.2.4. Influence of the Obtained Nanosystems on the Selection Rate of Persister Cells by Ciprofloxacin

We grew the microbial cultures for 4 h in the presence of 5 μg/mL (PAO1 laboratory strain and susceptible strains P.a 2, P.a 3, and P.a 7), or 15 μg/mL (for the antibiotic resistant strains P.a 1, P.a 4, P.a 5, P.a 6, P.a 8, and P.a 9) of ciprofloxacin, following an adapted protocol, previously described by [28], to obtain persister cells.

**Figure 8:**
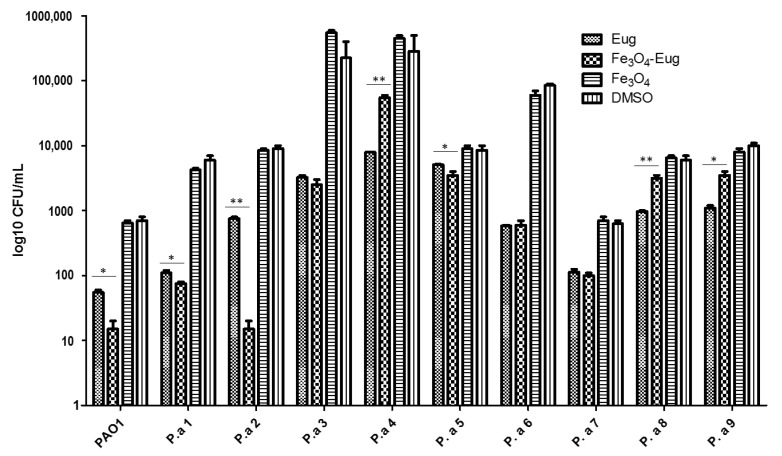
Graphic representation of the log_10_ values of colony-forming units (CFU)/mL representing persister cells selected after antibiotic exposure of *P. aeruginosa* strains, previously grown in the presence of functionalized nanoparticles and controls (one-way ANOVA, * *p* < 0.05, ** *p* < 0.001; significant evidence of the difference in CFU/mL values obtained by comparing eugenol and magnetite-eugenol nanoparticles samples).

The inhibition of persister cells production by the nanoparticles seems to be highly influenced by the antibiotic resistance/susceptibility profile of microbial strains. The most evident inhibition of persister cells selection was observed in the case of antibiotic susceptible PAO1, P.a 2, and P.a 7 strains, previously exposed to eugenol-functionalized magnetic NPs (Figure 8).

**Figure 9:**
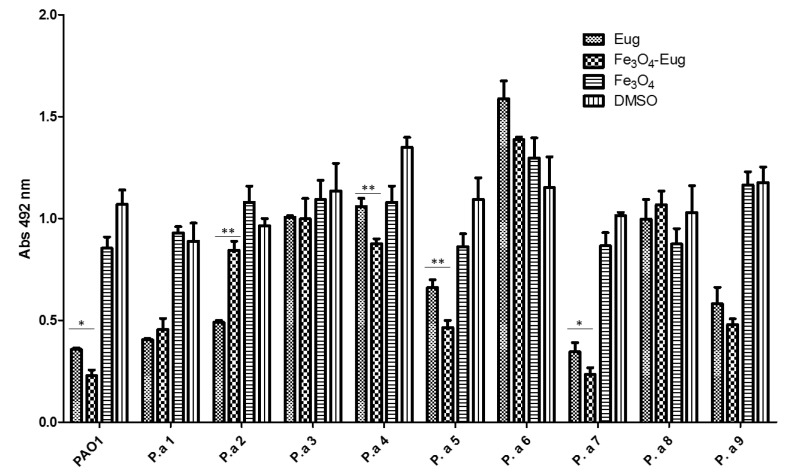
Graphic representation of absorbance values at 492 nm, which represent the optical density of *P. aeruginosa* biofilm-embedded cells, developed in the presence of sub-inhibitory concentrations of functionalized nanoparticles and controls (one-way ANOVA, * *p* < 0.05, ** *p* < 0.001; significant differences were obtained between eugenol and magnetite-eugenol nanoparticles).

Similarly, evidence of persister cells resulted in the case of antibiotic exposed *P. aeruginosa* biofilms, showing that the treatment with nanomaterials influences this phenotype differently, depending on the tested strain (Figure 9).

**Figure 10:**
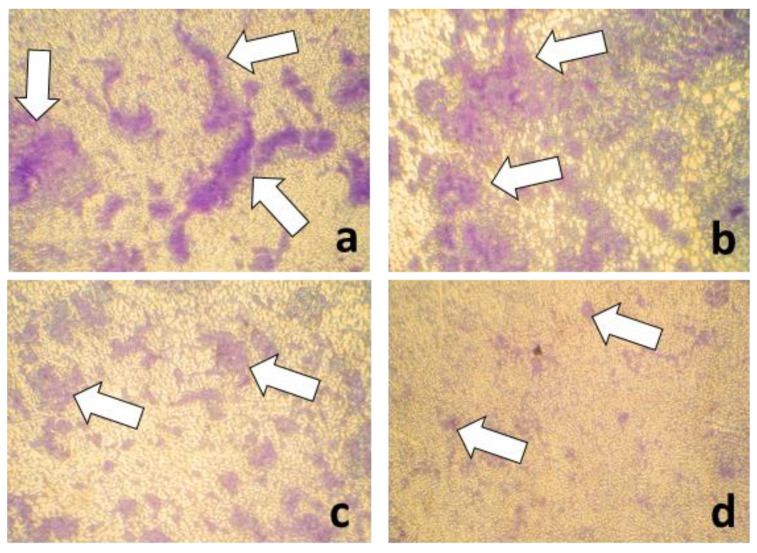
The aspect of biofilms produced by *P. aeruginosa* strain—PAO1 after cultivation in the presence of different nanomaterials/compounds tested for 24 h at 37 °C ((**a**) = untreated control, (**b**) = ciprofloxacin control, (**c**) = eugenol, (**d**) = Fe_3_O_4_-eugenol) (reversed microscopy, 400× magnification, Crystal violet staining). White arrows indicate cellular clusters suggesting biofilm development in respective areas.

We used a semi-quantitative method to quantify the biofilms mass, but it is not able to provide significant information regarding the cellular viability and metabolic status of biofilm cells. However, the microscopic analysis of the biofilm structure (Figure 10), as well as spectrophotometric analysis, indicated that eugenol functionalized nanoparticles significantly inhibited the development of monospecific biofilms.

## 3. Discussion

Nanotechnology finds nowadays applications in almost all fields of human activity, including pharmaceutical and biomedical ones. Various nanomaterials are used to fight planktonic bacteria and biofilms due to their intrinsic antibacterial and anti-pathogenic potential, as well as to their capacity to be used as delivery systems for natural compounds or synthetic drugs [29].

Magnetite has long been artificially obtained, first through the Massart method [30] in the form of a ferrofluid (colloidal liquid consisting of ferro/ferromagnetic nanoparticles suspended in water/organic solvent). To extend the range of applications, different methods have been developed for obtaining Fe_3_O_4_-based nanomaterials with suitable particle size, shape, distribution, particle surface chemistry, and magnetic properties [31]. Structurally, Fe_3_O_4_ crystallizes in cubic systems. The magnetic properties of this material are due to the presence of four unshared electrons in the 3-D layer of Fe^2+^ ion, respectively to the existence of five unshared electrons in 3-D layer of the Fe^3+^ ion [31]. Magnetite nanoparticles possess high surface energy and thus tend to quickly aggregate; they are also susceptible to leaching, but these properties could be balanced through the synthesis process [16,23,32]. These inorganic nanoparticles are preferred in developing bioactive nanosystems, including antimicrobial agents (due to their multiple targets at the level of bacterial cell, inducing membrane lesions, oxidative stress, enzymatic inhibition, protein deactivation, and transcriptome changes), being useful for controlled release, magnetic field manipulation, and stability [16,23,31].

In this study, the synthesis of magnetite nanoparticles dispersible in water was performed using an adapted co-precipitation method, more frequently cited in recent studies, due to its safety and good reproducibility and ability to provide good particle size control [22]. We obtained Fe_3_O_4_ NPs with sizes of 10 to 15 nm and low aggregative potential, which were used to obtain hybrid nanosystems containing eugenol and to assess both its antimicrobial and anti-biofilm activity, as well as the interference with the selection of persister cells by ciprofloxacin.

The antimicrobial activity of eugenol against different pathogenic strains, including *P. aeruginosa*, is well known in the literature [33], as well as its positive interaction with different hydrophobic and hydrophilic antibiotics active against gram-negative bacteria, being shown to decrease the MIC of the combination by a factor up to 1000, probably by increasing the permeability of bacterial membrane [34]. Therefore, this compound can be used to reduce the use of antibiotics and therefore reduce their associated toxicity and slow down the emergence of antibiotic resistance. Moroever, many studies, including ours, report on the anti-biofilm and anti-virulence activity of eugenol [35] and suggest as a possible mechanism of action its binding to Quorum Sensing receptor(s) [36].

The obtained hybrid nanosystem proved antimicrobial activity when used in association with eugenol at MIC concentrations of 312–625 µg/mL, with these concentrations being lower than those recorded for eugenol and magnetite controls. However, the most notable result is represented by the ability of the hybrid nanosystem to modulate key microbial virulence features, such as attachment and biofilm development, but also the production of soluble virulence enzymes, when used in sub-inhibitory concentrations.

An important biological effect of the obtained nanosystems is represented by their interference with the formation of persister cells, which curently represent a great challenge for the biomedical world [24].

Persistent and difficult to treat infections have multiple causes. In the most cases, they are produced by resistant etiological agents, which harbor different resistance genes or have the ability to organize themselves in microbial multicellular communities, called biofilms, becoming highly tolerant to high amounts of antimicrobials. As a result of external pressure exhibited by antibiotics and other chemicals affecting the fitness of the microbial population in both planktonic and biofilm growth states, a subpopulation with a changed metabolism and different behavior that aims to resist to the stressor agent can be selected, despite the identical genotype of all bacterial cells of the exposed population. This behavior is considered an epigenetic switch and is called bacteria bistability [37,38,39]. The bacterial cells resisting the action of antimicrobial substances enter a slow metabolic state and are called persisters or persistent cells. The selection of persistent cells can occur in either antibiotic susceptible or resistant microbial populations following exposure to certain antibiotics; however, the selection rate may be influenced by the genetic resistance features of the respective microbial cells [40]. Persisters can withstand the action of antibiotics in both planktonic microbial cultures, but especially in biofilms, representing one of the major causes of anti-infectious therapy failure. After ceasing the selective pressure, the microbial cells could move from metabolic latency to metabolically active cells, capable of growth and active division, generating a new microbial population [41]. *P. aeruginosa* is recognized as a species with a high rate of producing persister cells under antibiotic pressure [28]. Our results show that eugenol functionalized NPs may reduce the selection rate of persister cells in the presence of high antibiotic concentrations (i.e., 5–15 μg/mL ciprofloxacin). The results of the evaluation of persister cell production show that the used nanomaterials may influence this phenotype, but the results are strictly influenced by the dose, the resistance profile of microbial cells, and the utilized antibiotic. Our results cannot be extrapolated for other antibiotics, because exposure time or culturing conditions may vary, mainly due to bacteria bistability [39,42] and other less-known factors. However, our results reveal that NPs could potentiate the effect of eugenol on persister population selection, highlighting the utility of hybrid nanosystems, based on natural products and inorganic NPs, to reduce the selection of resistant cells under antibiotic pressure. This field is currently under intense investigation, with different categories of alternative agents, such as antimicrobial peptides [28], and other natural agents, such as phenol-soluble modulins, being proposed [43].

Taken together, the results obtained, although preliminary, clearly reveal the ability of functional magnetite NPs to act not only as classical bactericidal or bacteriostatic agents, as previously demonstrated [16,44], but also as efficient virulence modulators. The idea of utilizing molecules to modulate microbial virulence and social behaviors, although not new, has been heavily investigated in recent years [41,45] as clinicians and researchers seek alternative solutions to deal with severe and persistent infections. The antibiotic pressure that occurs during the classical treatment of infectious diseases represents the main factor for the selection of resistant mutants, since they interfere with the fitness of microbial populations which are obliged to adapt [46,47]. For this reason, researchers are investigating alternatives to control the infection without killing bacteria, thus without interfering with population fitness, since this reduces the risk of selecting resistant cells. The evolution of the infectious process can be manipulated by controlling the virulence arsenal of the infectious agent, such as attachment, biofilm formation, and release of degradative enzymes or other soluble virulence factors [45,48]. In this context, nanostructured systems able to modulate microbial virulence, such as the reported magnetic NPs, in association with natural compounds that are highly biocompatible and well tolerated, could represent an efficient alternative in fighting microbial infections, especially those produced by resistant pathogens as *P. aeruginosa*. Moreover, such agents could be used as synergic factors for the improved antimicrobial activity of antibiotics—antimicrobial synergisms between antibiotics and plant-derived compounds have recently been reported [49,50]—but also between antibiotics and NPs [51,52].

## 4. Materials and Methods

### 4.1. Materials

All required materials—ferrous sulfate hydrated with 7 water molecules (FeSO_4_·7H_2_O), ferric chloride (FeCl_3_), 25% ammoniacal solution (NH_3_), and eugenol (EUG)—were purchased from Sigma-Aldrich, were of analytical purity, and did not required further purification. Magnetite nanoparticles functionalized with EUG (Fe_3_O_4_@EUG) were obtained by co-precipitation method.

Briefly, 2 g of FeSO_4_·7H_2_O and 1,2 g FeCl_3_ were disolved in 400 mL of deionized water through vigorous mixing by using a glass baguette to obtain the precursory solution. Then, 8 mL of 25% NH_3_ were mixed with 800 mL of deionized water and 500 µL of EUG were added in the resulting solution. The precursory solution was then added dropwise in the NH_3_-EUG solution under magnetic stirring. The obtained nanoparticles were repeatedly washed with 200 mL deionized water, dried at room temperature, and mantained at +4 °C.

### 4.2. Physico-Chemical Characterization 

The physico-chemical characterization of the hybrid magnetic bionanostructured systems was performed by TEM (Transmission electron microscopy), SAED (Selected area electron diffraction), XRD (X-ray diffraction), and thermogravimetric analysis.

#### 4.2.1. TEM and SAED 

To perform transmission electron microscopy analysis, a Tecnai ™ G2 F30 S-TWIN HR-TEM (FEI Company, Hillsboro, OR, USA) equipped with SEAD was used. Sample preparation consisted of dispersing the powders in ethanol followed by 15 min of sonication and successive dilutions of the suspensions to obtain small concentrations of the samples to be analyzed. The next step was to place the suspensions on a carbon-copper grid that was cooled for analysis. The microscope was set in transmission mode at 300 kV, with a resolution of 2 Å and line resolution of 1 Å.

#### 4.2.2. X-ray Diffraction (XRD) 

An X-ray diffraction analysis of nanomaterial powders was performed with a Panalytical Empyrean diffractometer (step size 0.02, time per step 1 s) at room temperature. For all analyses made, Cu Ka radiations with l = 1.541874 Å were used. Samples were scanned at a Bragg 2u angle between 10–80.

#### 4.2.3. The Thermal Analysis TG-DSC 

The thermal analysis TG-DSC for the precursors was performed with a Netzsch STA 449C Jupiter apparatus (Netzsch, Selb, Germany). The samples (approximately 40 mg) were placed in an open crucible made of alumina and heated with 10 K·min^−1^ from room temperature up to 900 °C, under the flow of 50 mL min^−1^ of dried air. An empty alumina crucible was used as reference [23].

#### 4.2.4. Fourier-Transform Infrared Spectroscopy (FT-IR)

A Nicolet 6700 FT-IR spectrometer (Thermo Nicolet, Madison, WI, USA) connected to the software of the OMNIC operating system (Version 8.2; Thermo Nicolet, Madison, WI, USA) was used to obtain FT-IR spectra of the modified wound dressings. The samples were placed in contact with attenuated total reflectance (ATR) on a multibounce plate of ZnSe crystal at controlled ambient temperature (25 °C). FT-IR spectra were collected in the frequency range of 4000–650 cm^−1^ by co-adding 32 scans and at a resolution of 4 cm^−1^ with strong apodization. All spectra were ratioed against a background of an air spectrum.

### 4.3. Antimicrobial Activity Testing

#### 4.3.1. Bacterial Strains 

*P. aeruginosa* strains (1 laboratory [PAO1] and 9 clinical isolates, abbreviated P.a 1–P.a 9) were maintained on the nutrient broth with 20% glycerol at −80 °C. Among the selected clinical strains, two of them, P.a 2 and P.a 7, respectively, were susceptible to the majority of currently tested antibiotics for *P. aeruginosa*, while the rest of clinical isolates have shown antibiotic resistance or even MDR phenotypes [26]. For antimicrobial tests, the microorganisms were seeded on nutrient agar and Cetrimide selective media. The grown colonies were used to obtain 0.5 Mc Farland suspensions (1.5 × 10^8^ UFC/mL) in sterile physiological water.

#### 4.3.2. Antimicrobial Qualitative Assessment

The qualitative assay was performed using an adapted antibiotic susceptibility test following the procedure described in CLSI 2020. Briefly, a 0.5 McFarland microbial suspension (1.5 × 10^8^ CFU/mL) prepared in sterile saline water (0.9% NaCl solution) was swab inoculated on nutritive agar in a Petri dish. A total of 5 µL of each of the tested nano-suspensions (5 mg/mL) and controls were then added. The prepared dishes were incubated for 20 h at 37 °C. DMSO was utilized as stock solution of 50% concentration to respect the concentration found in the stock eugenol compound (prepared as 50% solution in DMSO). After incubation, the diameter of growth inhibition developed around the nano-suspension drop was measured and expressed in mm.

#### 4.3.3. The MIC (Minimum Inhibitory Concentration) Assay

A quantitative method based on binary serial microdilutions in nutrient broth distributed in 96-well plates was used to establish the MIC value. A concentration of 5 mg/mL of each tested compound/nanosystem was added to the first well of each row. Subsequently, binary dilutions were achieved by using a micropipette, starting from well 1 (concentration 5 mg/mL) to well 12 (where the final concentration will be 0.002441406 mg/mL). After the microdilutions were performed, 15 μL of 0.5 McFarland density microbial suspensions were added to each well. The plates were incubated for 24 h at 37 °C, and after incubation, the MIC value for compound/nanosystems was determined by visual examination as the lowest concentration at which no microbial growth was observed (lack of turbidity), and then confirmed by reading the absorbance of microbial culture at 600 nm using a spectrophotometer.

#### 4.3.4. Evaluation of Soluble Virulence Factors Expression

In order to reveal the production of some soluble virulence factors, we have utilized nutritive agar enriched with different ingredients in order to highlight the production of some enzymes capable of degrading/utilizing/modifying the respective substrate (e.g., for the detection of caseinase, we used casein-supplemented medium, gelatin for gelatinase, 5% blood-supplemented medium for hemolysins, Tween 80 medium for lipase, egg yolk medium for lecithinase, DNA-medium for DNA-se, etc.). Prior to cultivation on the mentioned media, microorganisms were cultured in liquid nutrient medium in the presence of sub-inhibitory concentrations of the hybrid nanosystems/controls for 4 h. After the inoculation on the specific media, the plates were incubated (24–72 h at 37 °C) and a medium modification (i.e., precipitate, the appearance of an opaque area or a transparent halo around the microbial colonies) was interpreted as a positive reaction [27].

#### 4.3.5. Evaluation of Persister Cell Selection after Antibiotic Pressure in Planktonic Cultures and Biofilms

A quantitative method was used, using liquid medium distributed in 96- multi well plates. After distributing the medium, the hybrid nanosystems/controls (MIC/2 value obtained for each strain) were added and the medium was inoculated with ~10^7^ CFU/mL of suspension prepared from the respective *P. aeruginosa* strains. The plates were incubated at 37 °C to allow the growth of microorganisms, and when they reached the late exponential growth phase (~12 h), the antibiotic ciprofloxacin (5 μg/mL for laboratory strain and ciprofloxacin susceptible strains and 15 μg/mL for ciprofloxacin resistant clinical *P. aeruginosa* isolates) [28] was added and the cultures were allowed to grow for another 4 h. After incubation, from the obtained microbial cultures, serial 10-fold dilutions were made and then 10 µL of each dilution was seeded in triplicate on Cetrimide agar for quantification of CFU (colony forming units)/mL. To evaluate the impact of the subinhibitory concentrations of the obtained nanosystem on total biofilms mass, the biofilms formed on the plastic walls were quantified. For this, the planktonic cultures were removed and the wells were washed with sterile saline water to remove unattached cells and fixed with cold methanol for 5 min. After removing the methanol, the dried plates were stained with 1% crystal violet solution for 20 min. After staining, the excess dye was washed with distilled water and the dye incorporated into the biofilm cells formed on the wells was solubilized with a 33% acetic acid solution. The absorbance of the obtained suspensions was read at 492 nm by using a spectrophotometer and the obtained values were proportional to the amount of developed biofilm. Before acetic acid solubilization, stained biofilms were visualized using an inverted bench optical microscope (Zeiss).

### 4.4. Statistical Analysis

Biological results were analyzed using the one-way ANOVA repeated measures test. All statistical analyses were performed using GraphPad Prism Software, v. 5.03 (GraphPad Software, La Jolla, CA, USA, www.graphpad.com, accessed on 22 February 2021).

## 5. Conclusions

This study reports on the design and antimicrobial activity of magnetite nanoparticles functionalized with a plant-derived compound, eugenol. Although the antimicrobial effect of eugenol is well known, the study demonstrates that lower amounts of this natural compound may be utilized in order to develop functional nanoparticles for the treatment of persistent infections in the future. Designing antimicrobial drugs based on natural compounds embedded in nanostructured shuttles represents an ecological and efficient antimicrobial strategy.

Moreover, the fact that the designed nanosystems may interfere with some virulence behaviors suggests that they could be utilized in subinhibitory concentrations to limit the selection of resistant microorganisms and to decrease the intensity of clinical symptoms associated with the progression of the infectious process, without necessarily interfering with population fitness.

Our results show that the obtained nanoparticles are able to limit the selection of persistent *P. aeruginosa* cells caused by antibiotic pressure, this being the first study reporting the efficiency of natural compounds functionalized nanoparticles and microbial persistence.

## Figures and Tables

**Table 1:** Thermic effects and mass loss data for the obtained nanoparticles.

Sample	RT-120 °C	120–400 °C	400–900 °C	Residual Mass %	Endo I Weak Bonded Molecules Elimination	Exo II Magnetite to Maghemite	Exo III Maghemite to Hematite
Fe_3_O_4_-control	1.54%	2.07%	0.26%	96.12%	52.3/73.1	345.4	538.8
Fe_3_O_4_-eugenol	1.11%	1.95%	0.56%	96.37%	83.7	299.0	566.7

## Sample Availability

Samples of the obtained NPs and microbial strains are available from the authors.
